# Quality and safety in prehospital airway management – retrospective analysis of 18,000 cases from an air rescue database in Germany

**DOI:** 10.1186/s12873-024-01075-x

**Published:** 2024-09-02

**Authors:** Ulf Lorenzen, Hartwig Marung, Christine Eimer, Andrea Köser, Stephan Seewald, Marcus Rudolph, Florian Reifferscheid

**Affiliations:** 1 Department of Anesthesiology and Intensive Care Medicine, University Hospital Schleswig-Holstein, Campus Kiel, Kiel, Germany; 2 Faculty of Health Sciences, Institute for Safety of Patients and Health Professionals (ISPP), MSH Medical School Hamburg, Am Kaiserkai 1, 20457 Hamburg, Germany; 3 Department of Emergency Medicine, University Hospital Schleswig-Holstein, Campus Kiel, Kiel, Germany; 4 Institute for Emergency Medicine, University Hospital Schleswig-Holstein, Kiel, Germany; 5 Department of Anesthesiology and Intensive Care Medicine, University Medical Centre Mannheim, Mannheim, Germany; 6 German Air Rescue “DRF Stiftung Luftrettung gAG”, Filderstadt, Germany

**Keywords:** Airway management, Adverse events, Emergency medical service (EMS), Patient Safety, Air Rescue, Difficult Airway, Trauma, Cardiopulmonary Resuscitation

## Abstract

**Background:**

Prehospital airway management remains crucial with regard to the quality and safety of emergency medical service (EMS) systems worldwide. In 2007, the benchmark study by Timmermann et al. hit the German EMS community hard by revealing a significant rate of undetected oesophageal intubations leading to an often-fatal outcome. Since then, much attention has been given to guideline development and training. This study evaluated the incidence and special circumstances of tube misplacement as an adverse peri-intubation event from a Helicopter Emergency Medical Services perspective.

**Methods:**

This was a retrospective analysis of a German helicopter-based EMS database from January 1, 2012, to December 31, 2020. All registered patients were included in the primary analysis. The results were analysed using SPSS 27.0.1.0.

**Results:**

Out of 227,459 emergency medical responses overall, a total of 18,087 (8.0%) involved invasive airway management. In 8141 (45.0%) of these patients, airway management devices were used by ground-based EMS staff, with an intubation rate of 96.6% (*n* = 7861), and alternative airways were used in 3.2% (*n* = 285). Overall, the rate of endotracheal intubation success was 94.7%, while adverse events in the form of tube misplacement were present in 5.3%, with a 1.2% rate of undetected oesophageal intubation. Overall tube misplacement and undetected oesophageal intubation occurred more often after intubation was carried out by paramedics (10.4% and 3.6%, respectively). In view of special circumstances, those errors occurred more often in the presence of trauma or cardiopulmonary resuscitation, with rates of 5.6% and 6.4%, respectively. Difficult airways with a Cormack 4 status were present in 2.1% (*n* = 213) of HEMS patients, accompanied by three or more intubation attempts in 5.2% (*n* = 11).

**Conclusions:**

Prehospital airway management success has improved significantly in recent years. However, adverse peri-intubation events such as undetected oesophageal intubation remain a persistent threat to patient safety.

**Trial registration:**

The study was registered in the German Register for Clinical Studies (number DRKS00028068).

## Background

Airway management in the emergency medical services (EMS) environment is often performed under difficult circumstances with respect to the severity of illness or trauma, impaired access to patients, time constraints and lack of information. These conditions may foster adverse events and pose a considerable threat to patient safety. Moreover, prehospital airway management success is often vitally important for long-term patient outcomes, i.e., survival. The 2007 benchmark study by Timmermann et al. served as a wake-up call not only to the German EMS community, making it perfectly clear that gaining competence in airway management but also to maintain that expertise is crucial for all EMS personnel. In their sample, undetected oesophageal intubation was associated with a 70% mortality [1]. As the German EMS system is highly reliant on emergency physician involvement, this aspect is of greatest importance to doctors working in that field.

The aim of this study was to evaluate trends in the efficacy and safety of prehospital airway management within the German Helicopter-based Emergency Medical Services (HEMS). The primary outcome was the incidence of adverse events in the form of endotracheal intubation failure, such as oesophageal intubation. The main hypotheses were as follows:

- The incidence of adverse events has significantly decreased since the work of Timmermann et al. [1] was originally published.
- airway management performed by HEMS physicians is safe and effective even under difficult Cormack-Lehane Grade III or IV anatomical conditions.

## Methods

A retrospective analysis of the German helicopter-based EMS association DRF Stiftung Luftrettung *(DRF)* database was conducted with regard to patients treated by HEMS physicians from January 1, 2012, through December 31, 2020. The database consists of comprehensive rescue mission data collected by 29 DRF helicopter bases in Germany. DRF helicopters are part of the German EMS system, serving as a support for ground-based responses by physicians and paramedics and covering emergencies in both adults and the pediatric population. The DRF medical crew consists of an emergency physician (mostly specialists in anesthesiology, surgery, or internal medicine) and a HEMS-TC (helicopter emergency medical system technical crew member trained as a paramedic). All medical personnel are formally qualified in terms of resuscitation and trauma care.

After arrival at a scene where endotracheal intubation had been performed by ground EMS staff, the position of the tube was instantly evaluated by the HEMS physician using end-tidal carbon dioxide or direct laryngoscopy as well as physical examination focused on the pulmonary status. When tube misplacement, such as oesophageal intubation, was encountered, the position was corrected immediately.

Data analysis in cases where intubation had been performed by HEMS physicians was based on electronic records containing a standardised set of items routinely collected during EMS missions, including basic patient demographics (e.g., sex, age), number of intubation attempts, and Cormack status.

Only emergency missions involving children less than 16 years of age and those involving the use of supraglottic airway devices were excluded from further evaluation.

The primary outcome measure was overall intubation success. Secondary measures consisted of Cormack status, the number of intubation attempts, the use of devices such as video laryngoscopy, and emergency diagnosis classification with respect to injury or other origin (non-trauma).

We used the STROBE checklist for observational studies to assess the strengths and weaknesses in the process of publication.

### Ethics approval and consent to participate

The study was approved by the Ethics Committee of the Medical Board of Baden-Württemberg, Germany (registration: F-2018-035). The need for consent to participate was deemed unnecessary according to German legislation (§ 27 Bundesdatenschutzgesetz, BDSG and § 89 Datenschutzgrundverordnung, DSGVO). Trial registration was performed retrospectively in the German Register for Clinical Studies (Deutsches Register Klinischer Studien, DRKS00028068).

Data collection was based on the emergency report (DIVI protocol) and the HEMSDER database (Convexis, Germany). Statistical analysis was performed using SPSS Statistics^®^ version 27.0.1.0. (IBM Corporation 2020, USA) and Microsoft^®^ Excel^®^ 2019 (Microsoft Corporation, USA). Crosstabs were processed and analysed by using a chi-square test. A probability value (P) of < 0.05 was considered to be significant.

## Results

**Fig. 1:**
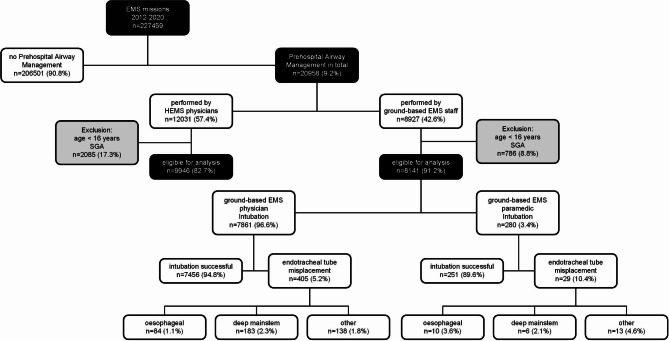
Inclusion and Exclusion Criteria

Out of 227,459 emergency medical responses, a total of 18,087 (8.0%) involved invasive airway management, i.e., endotracheal intubation. The detailed results can be found in the flow diagram “Inclusion and Exclusion Criteria” (Fig. 1). In 8141 (45.0%) of those patients, airway management was carried out by ground-based EMS staff. Most often, it was performed by ground EMS physicians (*n* = 7861), and in 280 cases, it was performed by paramedics, for an overall endotracheal intubation rate of 94.7%. We excluded patients receiving supraglottic airway (SGA) devices from the primary analysis. Most of the SGA used here were laryngeal tubes (*n* = 242, 2.9%). The use of combi tubes and laryngeal masks was rare, with 25 and 18 cases, respectively. The demographic data are presented in Table 1.

**Table 1:** General characteristics of the sample

	Overall EMS missions ^*^	Prehospital Airway management in total ^**^	Airway management by ground-based EMS ^**^	Airway management by HEMS physicians ^**^
**No. of cases**	**227,459**	**18,087**	**8141**	**9946**
Age: mean (SD)	56.2 (25.1)	57.2 (20.3)	56.8 (20.6)	57.6 (20.1)
Gender				
male (%)	131,093 (57.7%)	12,232 (67.6%)	5436 (66.8%)	6796 (68.3%)
female (%)	91,091 (40.0%)	5361(29.6%)	2464 (30.2%)	2897 (29.1%)
not specified (%)	5275 (2.3%)	494 (2.7%)	241 (3.0%)	253 (2.5%)
**Type of EMS mission**				
trauma (%)	86,062 (37.8%)	10,079 (55.7%)	4728 (58.1%)	5344 (53.7%)
non-trauma (%)	141.397 (62.2%)	8008 (44.3%)	3413 (41.9%)	4602 (46.3%)

^*^ excluded: age < 16

^**^ excluded: age < 16 and supraglottic airway devices

Overall, the rate of endotracheal intubation success was 94.7% (*n* = 7707), while tube misplacement was present in 5.3%, with a 1.2% rate of undetected oesophageal, a 2.3% rate of deep mainstem bronchus intubation and other inappropriate tube positions such as shallow intubation in 1.8% (Table 2). Overall tube misplacement and oesophageal intubation were more common after intubation by paramedics (10.4% and 3.6%, respectively) than by EMS physicians (5.2% and 1.1%), and this difference was statistically significant (*p* = 0.001).

**Table 2:** Intubation success by ground-based EMS

Total	8141	100.0%
**Correct tracheal tube position**	**7707**	**94.7%**
**Tracheal tube misplacement**	**434**	**5.3%**
Deep mainstem	189	2.3%
Oesophageal	94	1.2%
Other inappropriate tube position, e.g. shallow intubation	151	1.8%

Adverse airway management events occurred more often in the presence of trauma or CPR. Tube misplacement was found in 5.1% (*n* = 108 out of 2137) of trauma patients and 5.9% (*n* = 219 out of 4940) of CPR patients, although these differences were not statistically significant.

In 9946 of the 12,031 patients treated by HEMS (82.7%), endotracheal intubation was performed by DRF HEMS physicians. Overall intubation success by HEMS physicians was 100%, and first-pass success (FPS) was 86.9%, with a 96.3% FPS in Cormack 1 patients compared to 33.3% in Cormack 4 patients. Difficult airways, defined by a Cormack 4 status, were present in 2.1% (*n* = 213) of patients, 5.2% (*n* = 11) of whom required more than three intubation attempts (Table 3). The use of video laryngoscopy was documented in 5.7% of patients (*n* = 563, Tables 4 and 5). Intermediate ventilation via a bag valve mask was required in 28.5% (*n* = 2831) of patients, and intermediate ventilation via a laryngeal mask or laryngeal tube was required in 1.0% (*n* = 100) of patients.

**Table 3:** Number of endotracheal intubation attempts performed by DRF HEMS physicians subject to Cormack status

	1 attempt	2 attempts	3 attempts	> 3 attempts	total
Cormack 1	5666	203	14	2	**5885**
	96.3%	3.4%	0.2%	< 0.1%	**100%**
Cormack 2	2283	409	32	9	**2733**
	83.5%	15.0%	1.2%	0.3%	**100%**
Cormack 3	626	386	92	11	**1115**
	56.1%	34.6%	8.3%	1.0%	**100%**
Cormack 4	71	99	32	11	**213**
	33.3%	46.5%	15.0%	5.2%	**100%**
**Total**	**8646**	**1097**	**170**	**33**	**9946**
	**86.9%**	**11.0%**	**1.7%**	**0.3%**	**100%**

**Table 4:** Number of endotracheal intubation attempts subject to Cormack status and use of videolaryngoscope

	1 attempt	2 attempts	3 attempts	> 3 attempts	total
Cormack 1	223	20	2	0	**245**
	91.0%	8.2	0.8%	0.0%	**100%**
Cormack 2	129	33	5	2	**169**
	76.3%	19.5%	3.0%	1.2%	**100%**
Cormack 3	42	43	25	2	**112**
	37.5%	38.4%	22.3%	1.8%	**100%**
Cormack 4	7	14	9	7	**37**
	18.9%	37.8%	24.3	18.9%	**100%**
**Total**	**401**	**110**	**41**	**11**	**563**
	**71.2%**	**19.5%**	**7.3%**	**2.0%**	**100%**

**Table 5:** Endotracheal intubation aids by HEMS Physicians

Total	9946	100.0%
Videolaryngoscopy	563	5.7%
Guide	3014	30.3%
Ravuissan-/Cook-Catheter	19	0.2%
Rigid optic	1	0.0%
Fiber optic	5	0.1%
Not specified	6344	63.8%

## Discussion

### General findings

The German guidelines for prehospital airway management [2] emphasise that in emergency situations, direct laryngoscopy can be unexpectedly difficult even for experienced users [1] and calls for the highest first intubation success achievable [3, 4]. With an 8% rate of invasive airway management in the study sample, the accuracy of this statement is impressive.

Serious peri-intubation events of undetected false intubations are a persistent threat to patient outcomes, particularly for critically ill or injured sufferers. This observation is consistent with the findings of the INTUBE Study investigators. In their large international multicentre cohort study of critically ill adult patients requiring in-hospital airway management, Russotto et al. reported a 5.6% rate of oesophageal intubation. Overall 28-day mortality was 30.5%, with a mortality of 37.7% in patients with a major adverse peri-intubation event and 24.6% without events [5]. To further diminish these threats, several measures, from guideline development for prehospital airway management to legislative measures such as the establishment of significantly more comprehensive paramedic training and more effective quality management activities by emergency medical authorities, have been implemented in Germany and achieved some success.

### Ground-based EMS versus HEMS

The investigation revealed a proportion of 1.2% of undetected oesophageal misplaced intubations in comparison to the benchmark study by Timmermann et al. with a rate of 6.7% and the research of Caruana et al. with a rate of 5.5% [1, 6]. The studies by Silvestri et al. and Wirtz et al. reported approximately 9% unrecognised misplaced intubations [7, 8]. The high incidence of 25% out-of-hospital unrecognised misplaced endotracheal tubes performed by emergency physicians reported by Katz et al. in 2001, with 27 out of 108 improperly placed endotracheal tubes, (18 of these in an oesophageal position) has not been found ever since then [9]. However, these benchmark studies justify an ongoing evaluation of primary success in (prehospital) airway management.

### Intubation Success/Difficult Airway incidence

In the sample studied here, the overall first-pass success rate (FPS) reported by HEMS physicians (86.9%) was lower than that reported by Angerman et al., Driver et al. and Burns et al. [10–12]. The overall intubation success rate of 100% is in accordance with other studies of anaesthesiologist-staffed emergency response, with ETI success rates between 98 and 100% [13–15].

A prospective observational study by Knapp et al. in 2021 revealed an 87.6% FPS rate and an overall success rate of 98.6%, which is fully consistent with the findings presented here [16]. An investigation by Helm et al. in 2006 and a review by Crewdson et al. in 2017 reported similar results [13, 17]. Pietsch et al. reported that prehospital airway management is rare for HEMS crews (4% of all cases), compared to 8% in our sample [18]. Reinert et al. reported greater intubation success in HEMS than in ground-based EMS and emphasised that FPS is associated with the visualisation of vocal cords [19]. Our study also revealed a difference in the overall intubation success rate between HEMS (100%) and ground-based EMS (94.7%). In contrast, Thierbach et al. reported an overall intubation success rate of 100% among EMS physicians [14].

These results underline the findings of the systematic review by Bernhard et al. that there is an association between multiple intubation attempts and complications, as represented by the lower FPS rate of 33.3% reported by HEMS physicians in Cormack 4 patients [3]. Similarly, Sakles et al. proposed from an emergency department background that high-quality performance during orotracheal intubation indicated by a high FPS is associated with a rather small overall incidence of adverse events (AEs), while with an increasing number of attempts, the incidence of AEs is growing substantially [4]. The same is true for the prehospital environment [15]. Knapp et al. described even more clearly in 2017 that with two or more intubation attempts, the rate of complications (e.g., hypoxia, aspiration, cardiovascular arrest) increased by a factor of four to seven [20].

Severe airway management errors, such as undetected oesophageal intubation, were significantly more common after paramedic intubation than after physician intubation. These findings suggest the importance of continuous airway management training for paramedic personnel compared to most EMS physicians, who may be more skilled in (difficult) airway management due to their clinical experience and ongoing practice.

### Special circumstances: trauma and cardiopulmonary resuscitation

In accordance with the results of Hossfeld et al., we identified an impairment of airway management success in trauma and CPR (5.1% versus 5.9%, respectively) [21]. The results of Brown et al., who reported a lower FPS in medical versus trauma patients (93.4% versus 90.3%), support our findings [22].

A prospective observational study by Lockey et al. highlighted the relevant shortcomings of airway management in 472 trauma patients, with more than half of the sample initially treated by paramedic teams having significant airway compromise upon the arrival of an advanced care team. Major complications included failed tracheal intubation, unrecognised oesophageal intubation, and failure to administer oxygen [23]. We were not able to reproduce those results due to the systematic differences associated with paramedic-based and physician-staffed EMS systems.

According to the work of Jung et al., survival to discharge was significantly greater among patients who received ETI and SGA than among those who received BVM [24]. Nevertheless, the results from our sample suggest that airway management under these circumstances is especially prone to errors and should be performed by the most skilled member of the emergency team.

### Video laryngoscopy

In our sample, the overall first past success (FPS) rate following the use of a video laryngoscope was 71.2%. In patients with a Cormack 1 status, the FPS was 91.0% versus 18.9% in patients with a Cormack 4 status. Hossfeld et al. investigated 228 emergency patients who were receiving out-of-hospital airway management. HEMS physicians used C-MAC PM video laryngoscopy as a first-line device, which was associated with improved visualisation of the glottis and a success rate of 99.1% within a maximum of two attempts in out-of-hospital tracheal intubation [25]. Among 1417 in-hospital emergency patients, who were mostly treated by emergency medicine residents or critical care fellows, the first-pass success rate was 85.1% in the video laryngoscopy group and 70.8% in the direct laryngoscopy group [26]. In their Cochrane review of 222 studies, Hansel et al. reported that the use of video laryngoscopy results in higher success rates on the first attempt for adults and thus provides a greater level of safety than does direct laryngoscopy [27]. In the early phase of our study, the use of video laryngoscopy by HEMS physicians was not mandatory, and the respective standard operating procedure was subsequently modified. For that reason, our data are not fully comparable to those of the aforementioned papers. Since then, the availability of and training in video laryngoscopy have increased rapidly in German EMS systems.

### Limitations

Our study has several limitations, starting with the retrospective nature of the analysis of the HEMS database. As mentioned above, this could lead to a documentation bias. Rescue helicopters are dispatched as part of the German EMS system in selected cases (e.g., care for emergencies involving severely harmed patients, transport to specialised hospitals over long distances or multiple patients at the scene). Therefore, the study population does not represent the sample of emergency medical service missions in general. Furthermore, the airway expertise of HEMS physicians reported in our database, who were almost exclusively anesthesiologists, could have led to selection bias. In addition, we did not use the core dataset of an Utstein-style sample on prehospital airway management as recommended by Sunde et al. [28]. Follow-up data from hospitals, e.g., ICUs or 30-day mortality rates, were not available due to government restrictions and the large number of hospitals involved.

## Conclusions

The primary goal of this study was to evaluate trends with regard to adverse events in patients undergoing prehospital airway management within German Helicopter-based Emergency Medical Services (HEMS). These data represent the largest retrospective evaluation of airway management within the German EMS and thus provide comprehensive insight into the operational reality beyond clinical studies. The success rate of out-of-hospital airway management in Germany has increased significantly over the last 15 years. This means that the quality and safety of prehospital invasive airway management have improved. These encouraging results can be traced back to the fact that this issue has gained significant attention since Timmermann et al. published their results. Nevertheless, the rate of undetected oesophageal intubation remains a persistent threat with regard to patient safety. This is especially true for patients experiencing trauma and cardiopulmonary arrest. Based on the still inadequately high remaining risk of airway management errors described above, continuous efforts are necessary for further improvement of patient safety within the German EMS.

## Data Availability

The data presented in this study are available upon request from the corresponding author.
